# Fumarate-based drugs protect against neuroinflammation via upregulation of anti-ferroptotic pathways

**DOI:** 10.1186/s12974-025-03592-3

**Published:** 2025-10-27

**Authors:** Katinka Fischer, Leonie Thewes, Tim Prozorovski, Mary Bayer, Michael Dietrich, Torsten Lowin, Philipp Albrecht, Hans-Peter Hartung, Sven G. Meuth, Orhan Aktas, Carsten Berndt

**Affiliations:** 1 Department of Neurology, Medical Faculty and University Hospital Düsseldorf, Heinrich-Heine University, Moorenstr. 5, Düsseldorf, 40225 Germany; 2 Rheumatology, Medical Faculty and University Hospital Düsseldorf, Heinrich-Heine University, Düsseldorf, Germany; 3 Neurology, Kliniken Maria Hilf, Mönchengladbach, Germany; 4 Brain and Mind Centre, University of Sydney, Sydney, Australia; 5 Department of Neurology, Palacky University Olomouc, Olomouc, Czech Republic

**Keywords:** Inflammation, Multiple sclerosis, Vumerity, Therapy, Ferroptosis

## Abstract

Ferroptosis is defined as iron dependent non-apoptotic cell death. It is based on peroxidation of polyunsaturated phospholipids and subsequent membrane rupture. Lipid peroxidation is induced mainly by the hydroxyl radical, the product of the Fenton reaction between iron and hydrogen peroxide. Ferroptosis is connected to many organs and diseases including neuroinflammation. The most common inflammatory disease of the central nervous system in Western countries is multiple sclerosis leading to inflammatory demyelination and neurodegeneration. Fumarates, dimethyl fumarate (DMF) and its successor diroximel fumarate (DRF), are approved disease modifying therapies for multiple sclerosis and activate the Nrf2 pathway regulating the expression of many antioxidative enzymes. Since some of these enzymes are anti-ferroptotic, we here investigated whether the therapeutic effect of fumarates is connected to modulated sensitivity towards ferroptosis. Indeed, myelin damage induced by ferroptosis is diminished in presence of DRF or its active metabolite monomethyl fumarate. Moreover, anti-ferroptotic enzymes are upregulated in oligodendrocytes upon DRF treatment as well as in cerebellum of DMF-treated mice and in peripheral blood mononuclear cells of patients receiving DMF. This effect is absent in primary fibroblasts derived from osteo- or rheumatoid arthritis patients. In summary, our data offer a new organ- and disease-specific molecular anti-inflammatory mechanism of DRF.

**Supplementary Information:**

The online version contains supplementary material available at 10.1186/s12974-025-03592-3.

## Introduction

With around three million patients worldwide, multiple sclerosis (MS) is a common chronic inflammatory disease of the central nervous system characterized by focal demyelinating lesions in the brain and the spinal cord [1]. Since spontaneous remyelination is limited in the CNS and even rather suppressed in MS, demyelination often leads to axonal damage and, subsequently, neurological disability. The underlying inflammation is based on activated autoreactive T- and B-cells that cross the blood brain barrier [2–4]. MS occurs in different forms including relapsing-remitting (RRMS), primary progressive (PPMS), and secondary progressive (SPMS) MS. Although the knowledge of the disease and the number of drugs increased during the last 20 years, MS remains an incurable disease. Most approved therapies target inflammatory cascades to reduce relapse frequency and slow disease progression, prompting a search for non-canonical therapeutic targets [3]. Indeed, recently it was shown that ferroptosis might contribute to onset and progression of MS. Ferroptosis is an iron-dependent non-apoptotic cell death mechanism based on membrane rupture upon peroxidation of specific poly-unsaturated phospholipids [5, 6]. Increased Fenton reaction, the reaction between iron and hydrogen peroxide, is one of the main drivers of lipid peroxidation. In the mammalian system only glutathione peroxidase 4 (GPX4) is able to reduce the oxidized lipids to the corresponding non-toxic alcohols. Increased levels of iron and hydrogen peroxide, as well as decreased levels of GPX4 were described in MS patients and in the animal model experimental autoimmune encephalomyelitis (EAE) [7–12]. In addition, a marker for lipid peroxidation (4-hydroxynonenal, 4-HNE) was suggested as circulatory molecule to distinguish between RRMS and progressive MS [13]. Several inducers and inhibitors of ferroptosis are in use [6] and it was shown that pharmacological inhibition of ferroptosis mitigates EAE severity [14].

Here, we investigate whether the protective effects of diroximel fumarate (DRF) (and the predecessor molecule dimethyl fumarate (DMF)), two molecules used as drugs against RRMS (DRF: Vumerity^®^, DMF: Tecfidera^®^), are associated with induction of anti-ferroptotic mechanisms.

## Materials and methods

### Animal and patient material

Blood samples of patients with relapsing-remitting multiple sclerosis were collected for the biobank of the Department of Neurology, University Clinics Düsseldorf, Germany. This biobank is approved by the local ethics committee (Registration Number: 2017044238, Study Number 5951R, date of ethics vote: 28.06.2018). PBMCs isolated from twelve patients with an average age of 42 ± 10 years (4 male, 8 female) and an expanded disability status scale (EDSS) of 2.9 ± 1.9 or from healthy donors with an average age of 39 ± 10 years (2 male, 2 female) were investigated. The arthritis synovial fibroblasts were extracted from knees of 71 ± 5.6 (osteoarthritis (OA), 2 male, 1 female) or 82 ± 4 (rheumathoid arthritis (RA), 1 male, 3 female) years old patients for the RHINEVIT biobank of the Clinics for Rheumatology, University Clinics Düsseldorf, Germany, approved by the local ethics committee (2022–2189_7). The patients had CRP values of 14.7 ± 0.5 (OA) or 13.1 ± 1.3 (RA) mg/l, haemoglobine and erythrocyte sedimentation rates between 1 and 44.7 mm/h. Brains of DMF-treated mice came from a study published before [15]. Briefly, 30 mg/kg bodyweight of DMF (dissolved in DMSO) was administered daily for one week to 6 weeks old C57BL6/J female mice via the drinking water. Brains were dissected, snap-frozen and stored at −80 °C. For organotypic slice cultures, C57BL6/J pubs were used as approved by the Landesamt für Natur, Umwelt und Verbraucherschutz Nordrhein-Westfalen (LANUV) (O74/08) according to the German animal protection law (TierSchG).

### Organotypic slice culture

Preparation of cerebellar organotypic slice cultures was performed according to [16]. C57Bl/6J pups (postnatal days 9–11) were sacrificed by decapitation. The cerebellum was dissected and sectioned into 400 μm thick sagittal slices using a McIlwain Tissue Chopper (GaLa Instrumente). Organotypic slice cultures (OSCs) were dissociated in ice-cold dissecting medium (Hank’s Balanced Salt Solution (HBSS), Gibco) complemented with penicillin/streptomycin (100 U/ml, Gibco), 2.5 mg/ml glucose (Gibco) and 1 mM kynurenic acid (Sigma Aldrich). After 10 min of washing in ice-cold washing medium (50% MEM (Gibco); 47.5% HBSS, 2.5% HEPES), slices were cultured on Millicell-CM culture plate inserts (Millipore) in 50% MEM supplemented with penicillin/streptomycin (100 U/ml), 23.9% HBSS, 25% heat inactivated horse serum (Gibco) 1.1% Glucose, and 1% GlutaMAX (Gibco) in a humidified atmosphere with 5% CO_2_ at 35 °C. After 4 days in vitro slices were pretreated with 4 or 20 µM of fumarates or 200 nM liproxstatin 1 (LIP-1, provided by Marcus Conrad, Helmholtz Zentrum Munich, Germany) for 24 h before erastin (Sigma-Aldrich) or ferric ammonium sulphate (Sigma-Aldrich) was added for 4 h to the slices in 75% Opti-MEM (Gibco), 23.9% HBSS, 1.1% Glucose. After that, fumarates, LIP-1, or Salicylic acid isonicotinoyl hydrazine (SIH, provided by Ulf Brunk, University of Linköping, Sweden) was added for another 24 h, the slices were fixed with 4% paraformaldehyde (PFA) and analysis of myelin damage was performed by immunofluorescence as described before [17] by two raters blinded to treatment (CB and KF, blinded by MB). The mean values of damaged myelin across all cerebellar branches were calculated for every slice. The interrater reliability of these means analyzed by the Pearson correlation coefficient was 0.8722 (mean), with a range of 0.8452–0.9053. As a coefficient exceeding 0.8 was deemed almost perfect, all performed experiments were employed for analysis.

### Cell culture

OLN93 cells representing rat oligodendrocytes [18] were maintained in DMEM with 1 g/l glucose (Gibco) supplemented with 10% FBS (Gibco) and penicillin/streptomycin (100 U/ml). Osteoarthritis (OA) and rheumatoid arthritis (RA) synovial fibroblasts (SF) were extracted from the knee joint capsules of patients suffering either of osteoarthritis or rheumatoid arthritis [19]. The cells were cultivated in RPMI 1640 medium (Gibco) containing 10% FCS, penicillin/streptomycin (100 U/ml), and 1% GlutaMAX. To induce inflammation, OA and RA cells were incubated with 10 ng TNFa/ml for 3 days. All cells were maintained in a humidified atmosphere with 5% CO_2_ at 37 °C.

## Cell viability

Cell viability was determined in a 96-well plate containing 3,000 OLN93 cells (seeded 16 h prior treatment) or 10,000 inflamed OA/RA cells (cultured overnight) per well. The cells were pre-treated for 3–24 h with 2 or 10 µM of fumarates and afterwards with increasing concentrations of erastin or cumene hydroperoxide (Sigma-Aldrich) for another 3–24 h to induce ferroptosis with or without presence of 100 nM LIP-1. Following this, cells were incubated with CellTiter-Blue^®^ reagent (Promega), diluted in a 1:6 ratio in culture medium, for 1 h at 37 °C and 5% CO_2_. Fluorescence (545/600 nm) was measured using the CLARIOstar plus plate reader (BMG Labtech, Germany).

### Detection of lipid peroxidation

A total of 120,000 OLN93 cells or 200,000 inflamed OA/RA cells per well were seeded in a 6-well plate and pre-treated with 2 µM DRF or MMF for 3–24 h, followed by an incubation with or without 10 µM erastin for 6 h at 37 °C and 5% CO_2_ to induce lipid peroxidation (OA/RA cells were not treated to investigate whether fumarates are able to diminish inflammation induced lipid peroxidation). Following this, the cells were washed with phosphate-buffered saline (PBS), and lipid peroxidation was stained using 1 µM BODIPY 581/591 C11 for 15 min at 37 °C and 5% CO_2_. Subsequently, the cells were washed with PBS and trypsinized. The cell suspension was centrifuged at 700 g for 10 min and the pellet was resuspended in 300 µl of PBS, which contained 0.5% bovine serum albumin (BSA) or 10% FCS and 2 mM ethylenediaminetetraacetic acid (EDTA). Flow cytometry analysis was performed using the Cytoflex flow cytometry (Beckman Coulter) and the BD FACS Canto II. After establishing the cell gate (FSC-A/SSC-A), FSC-A and FSC-H were utilized for doublet discrimination. Oxidized BODIPY was measured in the FITC detector channel (excitation laser 488 nm; 530/30 bandpass filter). Data analysis was performed using Kaluza or FlowJo Software.

### Immunohistochemistry

Fixed organotypic slices were permeabilized with 1.5% (v/v) Triton X-100 (Sigma-Aldrich) in PBS for 45 min at room temperature (RT). After washing with PBS and incubation in blocking buffer (5–10% normal goat serum (Thermo Fisher Scientific); 0.25% Triton X-100 in PBS, 45 min), slices were incubated with rat anti-MBP (Chemicon; 1:500) and rabbit anti-NF200 (Chemicon; 1:500) antibodies diluted in blocking buffer overnight at 4 °C. After washing, the slices were incubated for 2 h at RT with secondary antibodies (goat anti-rat Cy3-conjugated and goat anti-rabbit Cy2-conjugated (Millipore, 1:500). DNA was counterstained by Hoechst 33342 (Sigma-Aldrich). The membrane with slices were mounted using Immumount. Sections of OA/RA patients were incubated for 1 h in blocking solution at RT and incubated with a 4-hydroxynonenal (4-HNE) antibody (1:250 in blocking solution) (Bioss) overnight at 4 °C. After washing, the sections were incubated with a secondary peroxidase-conjugated antibody (1:100 in blocking solution) (Jackson ImmunoResearch) for 1 h at RT, washed again and incubated for 3 min with 3,3´-diaminobenzidine and chromogen solution (Agilent). The reaction was stopped by incubating the sections for 5 min in dH_2_O. The sections were co-stained with haematoxylin for 1 s, washed and dehydrated (70% EtOH, 80% EtOH, 96% EtOH, and 100% EtOH). Afterwards, the sections were incubated in xylene for 3 min and subsequently preserved with Entellan. Samples were analyzed either with a BX51 microscope (Olympus) or a BZ-X800 microscope (Keyence).

### Western blotting

Protein concentrations were determined by Bicinchoninic acid assay via the Protein Quantitation Kit (Interchim). Samples were diluted in 1x Laemmli protein sample buffer (Bio-Rad, USA) and subsequently incubated for 5 min at 95 °C. Proteins were separated using Mini-PROTEAN^®^ TGX™ gels (Bio-Rad) in the Mini-PROTEAN^®^ Tetra System (Bio-Rad). Afterwards, the proteins were transferred to a 0.2 μm nitrocellulose membrane using the Trans-Blot^®^ Turbo™ Transfer System (Bio-Rad) and detected by immunostaining with primary (rabbit anti-GPX4 (1:1000; Abcam) and mouse anti-β-actin (1:5000; Abcam)) antibodies overnight at 4 °C). The membrane was washed with PBS containing 0.05% Tween and incubated for 1 h at RT with fluorochrome-labeled secondary antibodies (Odyssee). The visualization process was facilitated by the ChemiDoc Imaging System (Bio-Rad). Band intensity analysis was performed using the ImageLab or ImageJ software.

### RNA isolation, cDNA synthesis, and real-time qPCR

The isolation of ribonucleic acid (RNA) from cells and tissues was conducted using TRIzol Reagent (Thermo Fisher Scientific, USA) in accordance with the manufacturer’s protocol. Human blood samples stored in PaxGene tubes were thawed at 4 °C and RNA isolation was performed using the Qiagen PaxGene RNA Blood Kit following the manufacturer’s protocol. 1.5–2 µg of total RNA determined by using a NanoDrop 2000 spectrophotometer (Thermo Fisher Scientific) was used for cDNA synthesis with High-Capacity cDNA Reverse Transcription Kit (Applied Biosystems). The synthesis reaction was performed in the Biometra T Gradient Thermocycler (Analytik Jena AG). The synthesized cDNA was subsequently diluted to a concentration of 1:3 in nuclease-free water and qPCR analysis was performed using an Applied Biosystems 7000 instrument or a QuantStudio 3 instrument (Thermo Fisher scientific) and the SYBRGreen Master Mix (Invitrogen). Ct values were used to quantify relative expression of single genes normalized to *ACTB* or *GAPDH*. The following primers were used: *HMOX1* (human, mouse, rat): 5´-GCCGAGAATGCTGAGTTCATG-3´, 5´-TGCAGCTCCTCAGGGAAGTAGA-3´; *GPX4* (human, mouse, rat): 5´-CCGATACGCTGAGTGTGGTT-3´, 5´-TCACCACGCAGCCGTTCT-3´; SLC7A11 (rat): 5´-GGCTGGTTTTACCTTCAACTTTGTT-3´, 5´- ACATAGCCAATGGTGACAATGG-3´; FTH1 (rat, human): 5´-GCCATCAACCGCCAGATC-3´, 5´-TCATGAGATTGGTGAAGAAAGTATTTG-3´; FSP1 (rat, human): 5´-GTCAGCCAGGGCCACTTG-3´, 5´-CCAGGCCTATGAGGACATGGT-3´; *ACTB* (rat): 5´-CCCGCGAGTACAACCTTCTTG-3´, 5´-AAGCCGGCCTTGCACAT3-3´; *ACTB* (human, mouse): 5´-GGCACCCAGCACAATGAAG-3´, 5´-GCCGATCCACACGGAGTACT-3. Primers for detection of *GAPDH* were from Qiagen (GeneGlobe ID QT00079247).

### Statistical analysis

Statistical analysis was performed using version 4.0.5 of the R statistics package or GraphPad Prism 9 (GraphPad Software). The Wilcoxon rank-sum test was employed to compare two independent groups (e.g., two different treatments), assessing whether the distributions of their observations differ significantly. The Wilcoxon signed-rank test was used for paired data, such as pre- and post-therapy results, to evaluate whether there was a significant difference between paired observations within the same group. For analyses involving more than two groups, the one-way ANOVA test (Dunnetts multiple comparison) or the Kruskal-Wallis test with the Dunn post hoc test and Dunn-Bonferroni correction was applied. Results of statistical tests are presented as p-values with a significance level of alpha equal to 0.05.

## Results

**Fig. 1:**
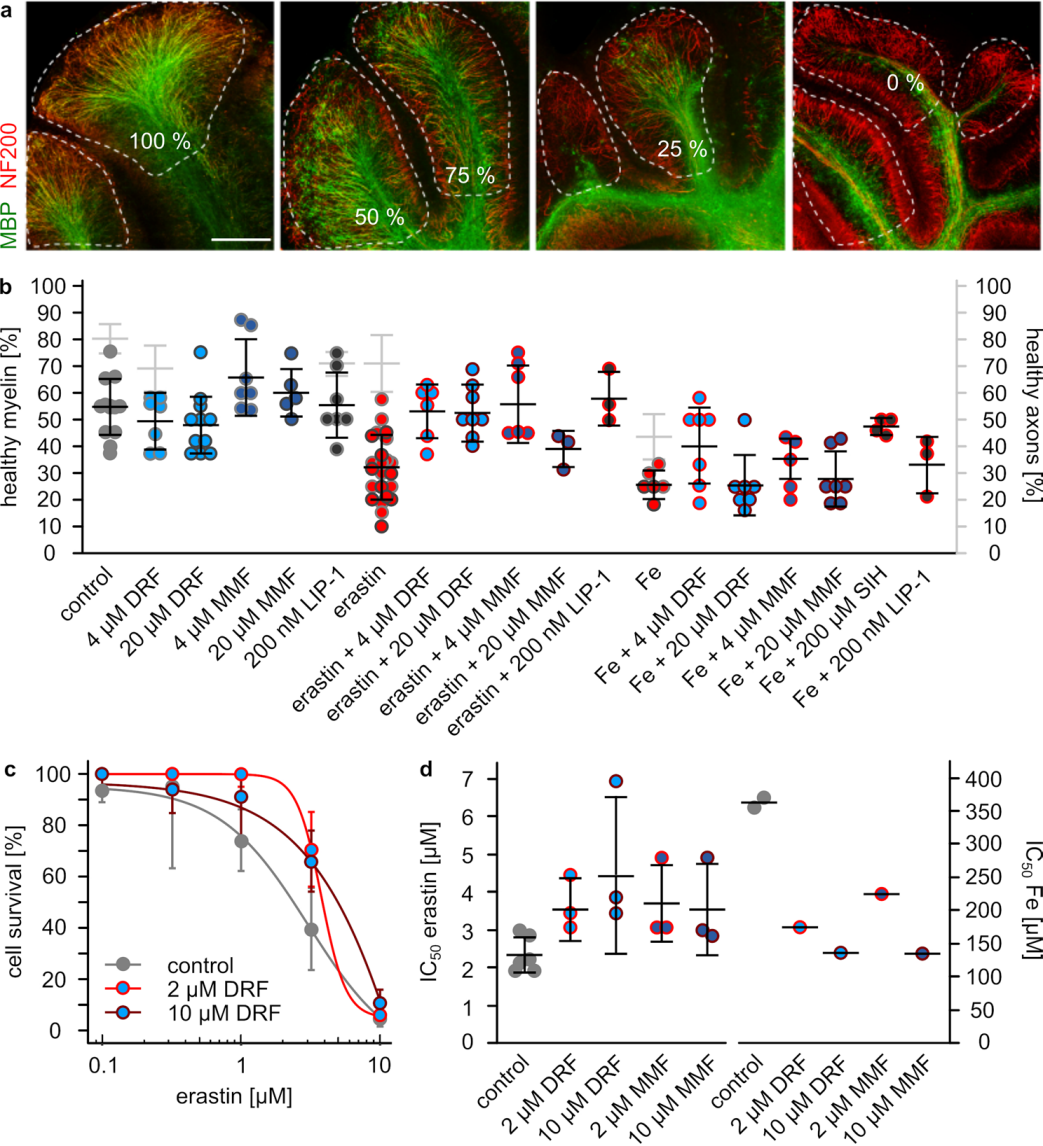
DRF and MMF protect myelin structure and oligodendrocytes against ferroptotic damage. **a** Immunohistochemistry showing myelin (anti-MBP antibodies) and axons (anti-Neurofilament 200 antibodies) in cerebellar slice cultures. Images demonstrate quantification of healthy myelin. **b** Quantification of healthy myelin structure (black) and axons (grey) in cerebellar slice cultures treated as indicated for 24 h with diroximel fumarate (DRF), monomethyl fumarate (MMF), liproxstatin 1 (LIP-1, a ferroptosis inhibitor), salicylic acid isonicotinoyl hydrazine (SIH, an iron chelator), and the ferroptosis inducers erastin and ferric ammonium sulphate (Fe). Every dot represents one organotypic slice culture, mean ± SD. **c** Cell survival (Cell Titer Blue) of OLN93 cells treated as indicated with fumarates and erastin, *n* = 3, mean ± SD. **d** IC_50_ values of OLN93 cells treated without or with 2 or 10 µM DRF or MMF for erastin based on c) and for iron (mean ± SD)

First, we assessed whether DRF and MMF can protect the myelin structure - the primary target during multiple sclerosis - from damage caused by ferroptosis. Therefore, organotypic cerebellar slice cultures from mouse brains were isolated, cultured and treated either with erastin or iron to induce ferroptosis. The percentage of healthy myelin was determined in control slices and slices incubated with DRF, MMF, the ferroptosis inhibitor liproxstatin 1 (LIP-1), or the iron chelator salicylaldehyde isonicotinoyl hydrazone (SIH) (Fig. 1a, b). The percentage of healthy myelin/OPC was similar in control slices (54 ± 11) and slices treated with DRF (4 µM: 50 ± 10%, 20 µM: 49 ± 11%) or LIP-1 (56 ± 13%), but slightly higher after MMF treatment (4 µM: 67 ± 14%, 20 µM: 60 ± 9%) (Fig. 1b). Treatment with 30 and 60 µM erastin dropped healthy myelin to 32 ± 12%. DRF (4 µM: 53 ± 10%, 20 µM: 53 ± 10%), MMF (4 µM: 56 ± 14%, 10 µM: 39 ± 7%), and 200 nM LIP-1 (58 ± 10%) were able to rescue the induced myelin damage (Fig. 1b). 1 and 3 mM iron decreased healthy myelin to 26 ± 5%. Here, only the iron chelator SIH showed a clear protective effect (47 ± 3% healthy myelin). Lower concentrations of DRF and MMF as well as LIP-1 led to some protection (41 ± 15, 35 ± 8, and 33 ± 11%, respectively), whereas higher concentrations showed no myelin preservation (20 µM DRF: 26 ± 11%, 20 µM MMF: 27 ± 11%). Iron obviously induced axonal damage in addition to myelin damage, whereas erastin did not. Treatment of slice cultures with 1 and 3 mM iron displayed 43 ± 9% healthy axons and 71 ± 11% after treatment with 30 and 60 µM erastin compared to 80 ± 5% in untreated control slices (Fig. 1b). Treatment with 4 µM DRF or 200 nM LIP-1 showed similar percentages of healthy axons (69 ± 8 or 71 ± 5) (Fig. 1b). To investigate whether DRF and MMF directy protect survival of oligodendrocytes against ferroptotic cell death, the cell line OLN93 (rat oligodendrocytes) was used. OLN93 cells were treated with increasing concentrations of either erastin or iron and the IC_50_ values for both compounds were determined in untreated control cells, and cells treated with 2 or 10 µM DRF or MMF (Fig. 1c, d). The IC_50_ values increased from 2.3 ± 0.5 µM erastin in control cells to 3.6 ± 0.7 (2 µM DRF), 4.4 ± 2.2 (10 µM DRF), 3.7 ± 1.0 (2 µM MMF), as well as 3.6 ± 1.2 µM (10 µM MMF), and a rough assessement with iron in the same control cells a decrease from 362.5 µM to 173 (2 µM DRF), 138 (10 µM DRF), 225 (2 µM MMF), as well as 135 µM (10 µM MMF), respectively (Fig. 1d).

**Fig. 2:**
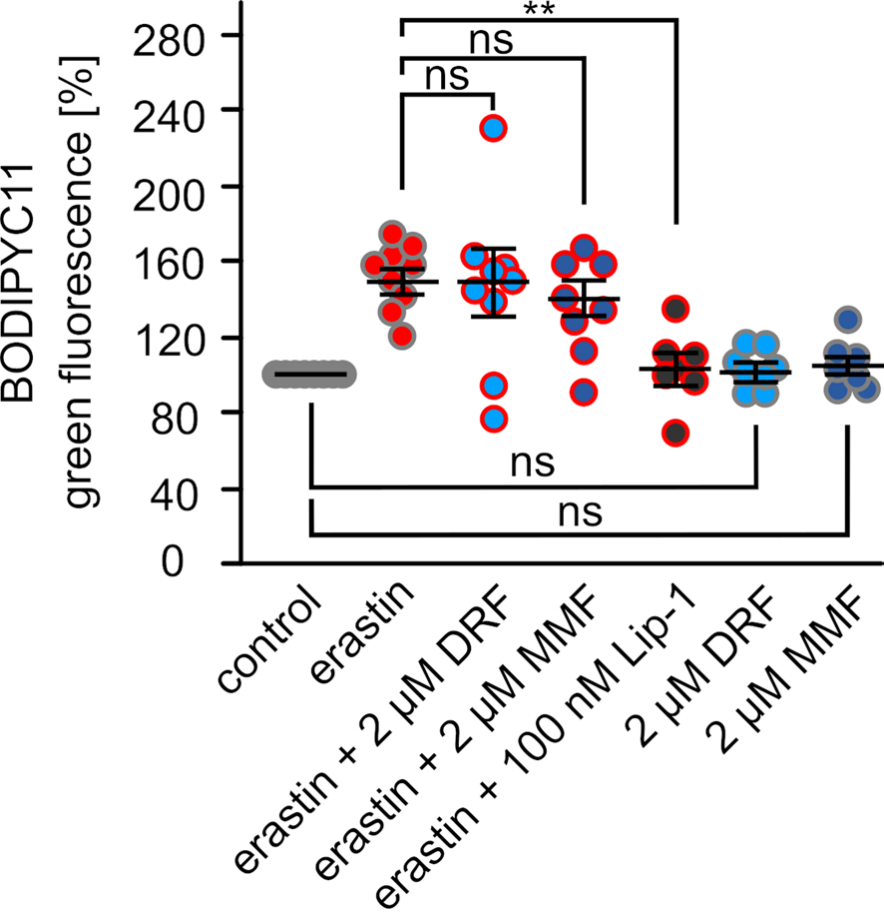
DRF and MMF show no significant effect on lipid peroxidation. Flow cytometry analysis of lipid peroxidation via BODIPY fluorescence in OLN93 cells treated with erastin, DRF, MMF, and the ferroptosis inhibitor liproxstatin-1 (LIP-1) as indicated. Every dot represents an independent experiment, mean ± SEM, significance was calculated using the Kruskal-Wallis test with the Dunn post hoc test and Dunn-Bonferroni correction, *: *p* < 0.05

The protective effect of DRF and MMF on erastin-induced myelin damage and oligodendrocyte survival seems not to be associated with decreased lipid peroxidation. In contrast to LIP-1 (104 ± 7% compared to untreated control cells), 2 µM DRF (151 ± 15%) or 2 µM MMF (139 ± 10%) were not able to diminish the erastin-induced lipid peroxidation (152 ± 5.7%) (Fig. 2). Treatment of cells just with 2 µM DRF (104 ± 7.5%) or MMF (102 ± 3.9%) showed also no protective effect against lipid peroxidation without ferroptosis induction (Fig. 2).

**Fig. 3:**
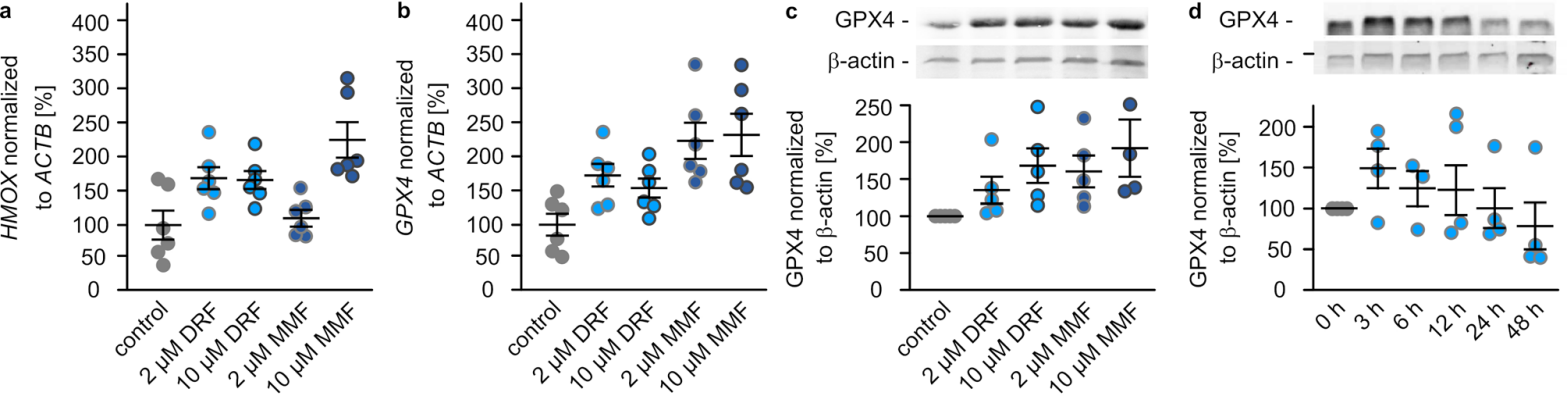
DRF and MMF increase GPX4 expression. qPCR of *HMOX* (**a)** and *GPX4* (**b)** using mRNA isolated from OLN93 cells treated with 2 or 10 µM diroximel fumarate (DRF) or monomethyl fumarate (MMF). Transcription levels were normalized to that of *ACTB* (β-actin). Each dot represents an independent experiment (mean ± SEM). **c** Western blot (picture of representative blot and quantification of 4–5 independent experiments, mean ± SEM) showing GPX4 and β-actin using protein extract isolated from OLN93 cells treated with indicated concentrations of DRF and MMF for 24 h. **d** Western blot (picture of representative blot and quantification of 4 independent experiments, *n* = 4) showing GPX4 and b-actin using protein extracts isolated from OLN93 cells treated with 2 µM DRF for 3–48 h

The predecessor molecule of DRF, DMF, acts rather as an oxidant than an antioxidant providing protection against oxidative damage by activating the antioxidative response via the Nrf2 (nuclear factor erythroid 2-related factor 2) pathway [20]. We confirmed that DRF and MMF activate the Nrf2 signaling measuring the mRNA levels encoding heme oxygenase 1 (HMOX), a marker protein of Nrf2 signaling. As shown in Fig. 3a, 2 (168 ± 16%) and 10 µM DRF (166 ± 13%) as well as 2 (111 ± 12%) and 10 µM of MMF (224 ± 26%) increased the levels of *HMOX* mRNA compared to untreated cells (100 ± 22). The relationship between Nrf2 and GPX4 - the only mammalian enzyme capable to reduce lipid hydroperoxides – has not been thoroughly investigated [6]. Nevertheless, we tested next whether DRF and MMF are able to increase expression of GPX4. Compared to untreated control cells (100 ± 16%), mRNA levels of GPX4 increased upon treatment with 2 (172 ± 17%) and 10 µM DRF (153 ± 14%), as well as 2 (223 ± 26%) and 10 µM MMF (232 ± 31%)(Fig. 3b). These data were confirmed on protein level (Fig. 3c). Again, DRF and MMF treatment increased GPX4 in OLN93 cells. This increase of GPX4 levels was visible after three hours of treatment (150 ± 25% compared to untreated cells) and lasted for approximately nine additional hours (6 h: 124 ± 23%, 12 h: 123 ± 34%)(Fig. 3d). 12 h after treatment GPX4 levels were back to control levels (101 ± 23%) and after 48 h it further decreased to 79 ± 33%) (Fig. 3d).

**Fig. 4:**
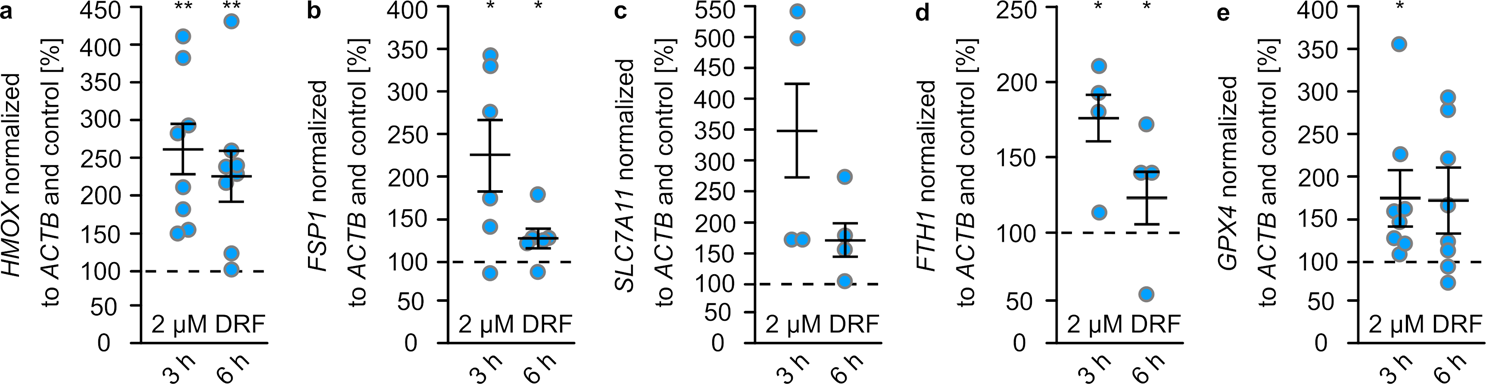
DRF increases transcription of genes encoding anti-ferroptotic proteins. Transcript levels of *HMOX* **(a)** *FSP1* (**b)** *SLC7A11* (**c**), *FTH1* (**d)** and *GPX4* (**e)** of OLN93 cells treated with 2 µM DRF for 3–6 h. Ct values of qPCRs were compared to untreated cells and *ACTB* levels for normalization. Significance was calculated using the Kruskal-Wallis test, *:*p* < 0.05, **:*p* < 0.01, mean ± SEM

We confirmed that this fast increase of HMOX and GPX4 was also reflected by mRNA levels. In addition, we measured the impact of DRF on additional anti-ferroptotic proteins with clear ARE (antioxidant response element)-dependent transcription, e.g., ferroptosis suppressor protein 1 (FSP1), system x_c_^−^ (SLC7A11), and ferritin (FTH1). All measured transcripts show a strong increase after 3 h of treatment (*HMOX*: 258 ± 36%, *FSP1*: 226 ± 43%, *SLC7A11*: 347 ± 102%, *FTH1*: 380 ± 206%, *GPX4*: 177 ± 29%, Fig. 4) and a lower increase after 6 h (*HMOX*: 232 ± 35%, *FSP1*: 132 ± 12%, *SLC7A11*: 178 ± 35%, *FTH1*: 137 ± 24%, *GPX4*: 176 ± 34%, Fig. 4).

**Fig. 5:**
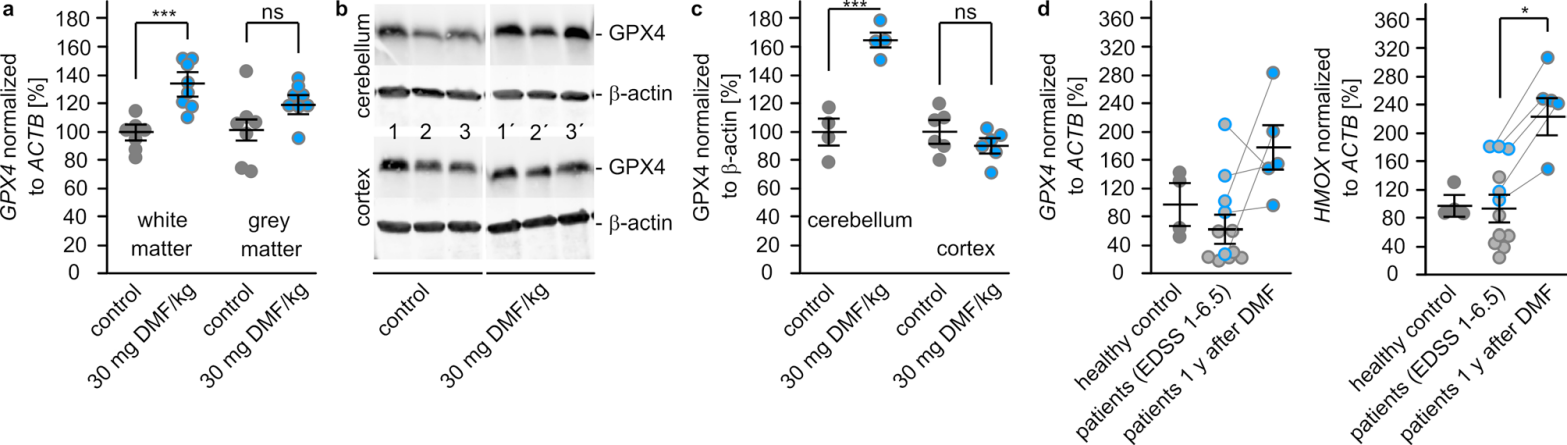
DMF increases GPX4 levels in brains of mice and blood of multiple sclerosis patients. **a** qPCR of GPX4 using mRNA isolated from white or grey matter of mice without or with DMF treatment (30 mg/kg for 7 days). Ct values of 7 individual mice were compared to the mean of control white matter and to *ACTB* for normalization. **b** Western blots of cerebellum and cortex using protein lysates isolated from 3 individual mouse brains without or with DMF (see a) showing GPX4 and β-actin. **c** Quantification of b, mean ± SEM). **d** qPCR of *GPX4* and *HMOX* using mRNA isolated from controls (no multiple sclerosis, grey dots) and multiple sclerosis patients before and after 1 year of DMF treatment (blue dots). Ct values were compared to the mean of controls and *ACTB* for normalization. Significance was calculated via the Wilcoxon signed-rank test, *: *p* < 0.05, ***: *p* < 0.001, mean ± SEM

Next, we aimed to translate our results to animals and patient samples. Here, we used available samples from mice and patients treated with DMF. qPCR with mRNA isolated from white and grey matter of mice brains treated with 30 mg DMF/kg for 7 d showed a significant increase of GPX4 encoding mRNA in white (136 ± 7%), but not in grey matter (120 ± 6%) compared to the respective brain regions without treatment (100 ± 4% in white, 100 ± 8% in grey matter) (Fig. 5a). This finding was confirmed by western blots using cell extracts of cerebellum and cortex of the same mice (Fig. 5b). Whereas samples from cerebellum displayed a significant increase in GPX4 protein (164 ± 6% compared to cerebellum without DMF treatment), samples from cortex did not show higher GPX4 levels (90 ± 5% compared to cortex without DMF treatment (Fig. 5c).

To see whether DMF treatment affects GPX4 levels in RRMS patients we selected PBMC samples from the biobank of the Department of Neurology. Twelve patient samples with an average age of 42 ± 10 years (4 male, 8 female) and an expanded disability status scale (EDSS) of 2.9 ± 1.9 were measured for *GPX4* and *HMOX* transcripts and compared to PBMCs isolated from healthy donors with an average age of 39 ± 10 (2 male, 2 female). In samples from RRMS patients we observed a downregulation of *GPX4* mRNA to 65 ± 17% compared to healthy controls (100 ± 24%), whereas *HMOX* mRNA was not regulated in MS (96 ± 16%) (Fig. 5d). Five of the RRMS patients (age: 40 ± 11 (2 male, 3 female), EDSS: 1.7 ± 0.4) donated samples after one year of DMF treatment. DMF treatment increased the *GPX4* mRNA levels in 4 out of 5 patients, in total to 160 ± 28% (Fig. 5c). The amount of *HMOX* mRNA increased in all 5 treated patients significantly to 175 ± 19% (Fig. 5d).

**Fig. 6:**
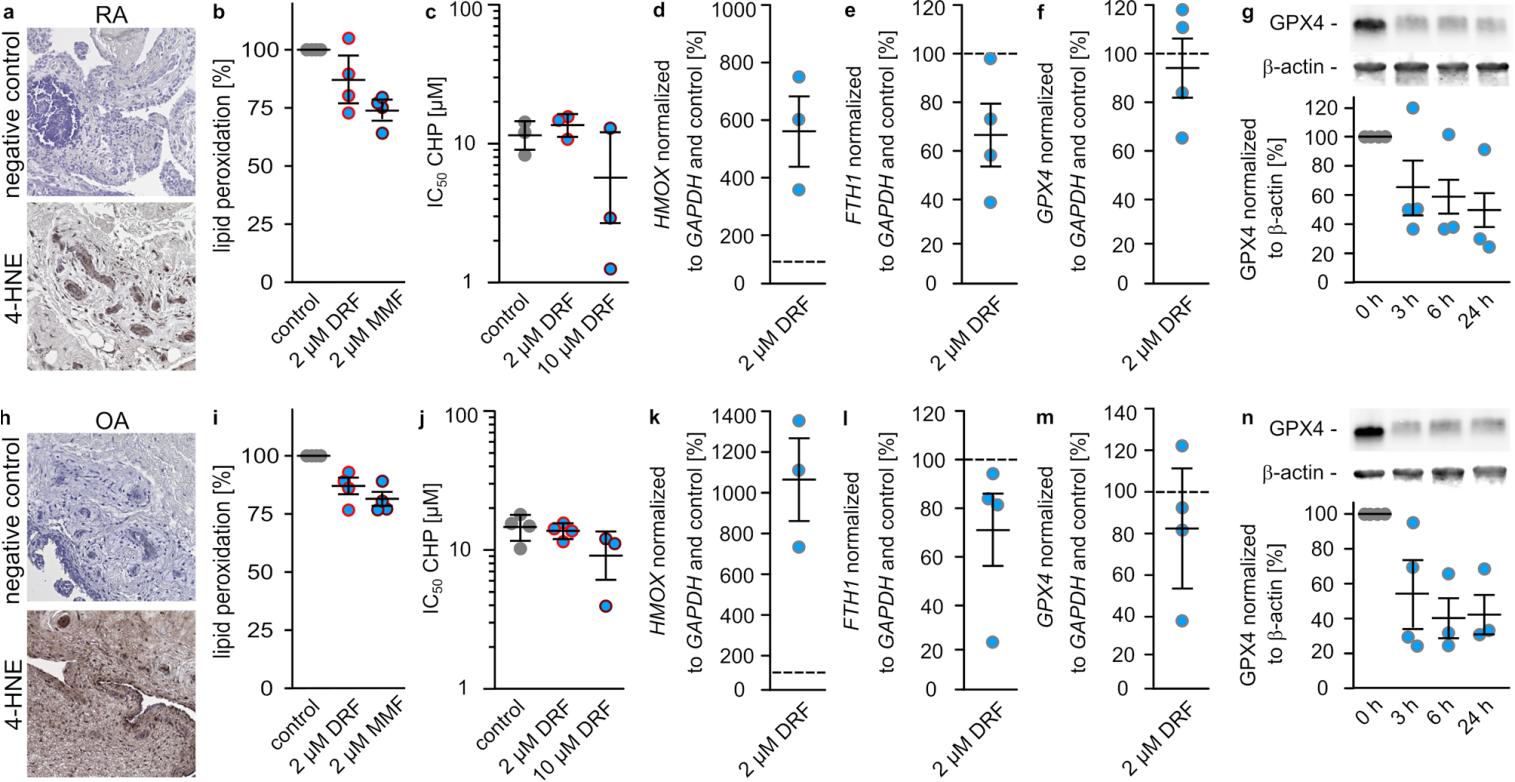
Impact of DRF on ferroptosis in rheumatoid arthritis (RA) and osteoarthritis (OA). **a** 4-HNE staining in synovial tissue of an RA patient. **b** Flow cytometry analysis of lipid peroxidation via BODIPY fluorescence ± 2 µM DRF or MMF of RA patient derived synovial fibroblasts after induction of inflammation using TNFα (mean ± SEM). **c** IC_50_ values calculated from survival assays of RA patient derived fibroblasts ± 2 µM DRF or MMF after TNFa treatment. **d**-**f** qPCR of indicated transcripts in RA patient derived fibroblasts with induction of inflammation via TNFα treated for 3 h with 2 µM DRF compared to untreated cells using *GAPDH* transcripts for normalization (mean ± SEM). **g** Protein levels of GPX4 in TNFα incubated RA patient derived fibroblasts treated with 2 µM DRF for indicated time points measured by western blotting using anti-GPX4-antibodies and anti-β-actin-antibodies for normalization. Representative staining and quantification (mean ± SEM) is shown. **h**-**n** Same experiments as a-g using OA patient-derived fibroblasts. Every dot represents an independent experiment

To reveal whether these findings are specific for autoimmune diseases in the central nervous system, we used rheumatoid arthritis (RA) and osteoarthritis (OA) as further disease models. RA is based on chronic inflammation in joint cartilage induced by auto-antibodies, whereas OA is a non-autoimmune degenerative disease of the same tissue. In tissues of RA and OA patients, lipid peroxidation was detected. Both, RA and OA samples show lipid peroxidation (Figs. 6a, h). However, DRF or MMF has only minor effects on lipid peroxidation (RA: 87 ± 6.8% after DRF treatment and 74 ± 3.4% after MMF treatment compared to untreated controls (Fig. 6b), OA: 85 ± 3.4% after DRF treatment and 80 ± 2.9% after MMF treatment compared to untreated controls (Fig. 6i) and survival (Figs. 6c, j) after induction of inflammation via TNFα in isolated primary fibroblasts cells from RA/OA patients. The IC_50_ value for CHP changed from 11 ± 1.7 µM in untreated cells to 14 ± 1.5 µM or 5.7 ± 3.6 µM after treatment with 2 or 10 µM DRF in RA cells (Fig. 6c) and in OA cells from 15 ± 1.5 µM to 14 ± 0.9 µM or 9 ± 3.6 µM (Fig. 6j). In line with previous presented data, the isolated fibroblast cells show an increase in *HMOX* mRNA (RA: 572 ± 105% (Fig. 6d), OA: 1077 ± 181% (Fig. 6k). However, in contrast to oligodendrocytes, fibroblasts do not show increased transcripts of *FTH1* (RA: 66 ± 13% (Fig. 6e), OA: 69 ± 16% (Fig. 6k, l) or *GPX4* (RA: 93 ± 13% (Fig. 6f), OA: 81 ± 19% (Fig. 6m) after treatment with DMF for 3 h. Reduced expression of GPX4 upon DRF treatment was confirmed on protein level as shown in Figs. 6g and n. In RA-based fibroblasts, 2 µM DRF decreased GPX4 to 65 ± 19% after 3 h, to 59 ± 21% after 6 h, and to 48 ± 21% after 24 h (Fig. 6g) and in OA-based fibroblasts to 55 ± 21% after 3 h, to 40 ± 11% after 6 h, and to 43 ± 12% after 24 h (Fig. 6n).

## Discussion

DRF, DMF, and MMF activate the transcription factor Nrf2 [21, 22] and thereby transcription of genes such as FSP1 and SLC7A11 [23] (Fig. 4). The gene encoding GPX4 lacks an ARE and is therefore most likely not regulated via Nrf2 [24]. However, here we show that DRF also induces upregulation of GPX4 transcripts and proteins in a time- and concentration-dependent manner (Fig. 3). Treatment of oligodendrocytes with DRF led to upregulation of GPX4 for 12 h, aligning with the twice-daily administration of the drug in multiple sclerosis patients. Consistently, GPX4 levels were elevated in PBMCs of patients after one year of DMF treatment (Fig. 5d). GPX4, FSP1, and SLC7A11, but also ferritin are all anti-ferroptotic proteins [6]. Ferroptosis is characterized as iron-dependent non-apoptotic cell death based on membrane rupture upon lipid peroxidation [5]. GPX4 is the only mammalian enzyme that can detoxify lipid hydroperoxides to the corresponding alcohols [6]. Since this enzyme needs GSH as cofactor, SLC7A11, the cystine importer guaranteeing high intracellular levels of GSH, supports the function of GPX4 [6]. FSP1 is able to reduce phospholipid peroxyl radicals to lipid hydroperoxides, whereas ferritin is able to bind iron, thereby limiting the amount of redox active free iron that can induce lipid peroxidation itself or via induction of Fenton reaction [6]. Ferroptosis is linked to multiple sclerosis and its animal model EAE as demonstrated by several publications. MS lesions show high lipid peroxidation [14] and MS patients as well as EAE mice display decreased levels of GPX4 and SLC7A11 [8, 9, 25, 26]. Moreover, inhibitors of ferroptosis diminish EAE [9, 14, 25]. We used an ex vivo model - organotypic slice cultures – to show that erastin, an inhibitor of SLC7A11, as well as increased iron levels induce myelin damage. DRF was able to rescue myelin structure after erastin treatment, but not after iron treatment (Fig. 1). Of note, liproxstatin 1 was also not able to abolish the effect of iron, only iron chelation was helpful (Fig. 1). Although ferritin expression was upregulated by DRF, the iron binding capacity was obviously not efficient, whereas the upregulated GPX4 expression was. The data obtained with OSCs indicate that oligodendrocytes are affected by ferroptosis. This is a bit in contrast to the published finding that GPX4 protein levels show no changes in oligodendrocytes but in neurons in EAE and multiple sclerosis patients [8]. However, other publications describe that ferroptosis inhibitors diminish oligodendrocyte/myelin loss induced by cuprizone [26] and that especially mature oligodendrocytes are sensitive against ferroptosis [27]. In addition, the cell culture data of the present study were obtained in an oligodendrocyte cell line (OLN93) to show the impact of fumarates on ferroptotic hallmarks.

In addition to multiple sclerosis, we wanted to know whether DRF can also protect against ferroptosis in the context of another autoimmune disease. DMF seems to protect against ferroptosis in a variety of diseases, including traumatic brain injury [28], liver ischemia-reperfusion [29], chronic cerebral hypoperfusion [30], or acute kidney injury [31]. We have chosen rheumatoid arthritis as another autoimmune disease as ferrostatin and liproxstatin 1 rescues RA and OA cells against inflammation [32] and iron-induced ferroptosis in a rheumatoid mouse model [33]. Moreover, DMF-activated Nrf2 signaling protects in the complete Freund’s adjuvant- induced arthritis model [34] and DMF diminishes apoptosis in RA cells isolated from patients [35]. As shown before [36], we confirmed lipid peroxidation in RA and OA patients (Fig. 6). However, to our surprise, DRF showed no upregulated expression of GPX4 in synovial fibroblasts obtained from RA and OA patients, GPX4 was rather downregulated (Fig. 6). This indicates that the protective effect of DMF on inflammation in RASF [35, 37] seems to be independent on ferroptosis and that fumarates act in a disease specific manner. As a fibroblast cell line derived from MS patients show increased lipid peroxidation and diminished GPX4 levels [38], we assume that the observed differences in DRF-mediated GPX4 levels do not depend on the different cell types used, fibroblasts and oligodendrocytes. The observation that fumarates induce molecules important for signaling events and triggering a beneficial response in lower concentrations but damaging in higher concentrations ([20] and this study) may also contribute to cell-type or disease-specific concentration-dependent therapeutic effects. This observation is consistent with one of the oldest theories in medicine, the principle of hormesis by Paracelsus (1493–1541) and demonstrated in Nrf2-dependent signaling pathways [39]. Indeed, Nrf2-dependent resilience against oxidative damage in neurodegeneration has been directly connected to the hormetic vitagene pathway [39, 40].

In conclusion, our data show that myelin structure is sensitive towards ferroptotic damage and that fumarates (DRF, DMF, MMF) can help to mitigate ongoing ferroptosis-related damage processes. This might be true for several autoimmune/inflammatory diseases, however, the mode of DRF action seems to differ. Moreover, in line with other publications, our data characterize ferroptosis as a relevant cell death mechanism in autoimmune disorders. In case of multiple sclerosis, ferroptosis might be a main driver of disease progression leading to oligodendroglial cell death in concert with other cell death mechanisms such as apoptosis and pyroptosis [41, 42].

## Supplementary Information

Supplementary material 1.

Supplementary material 2.

Supplementary material 3.

## Data Availability

All data generated or analyzed during this study are included in this published article.

## Abbreviations

ARE: Antioxidant response element
DMF: Dimethyl fumarate
DRF: Diroximel fumarate
EAE: Experimental autoimmune encephalomyelitis
GPX: Glutathione peroxidase
4-HNE: 4-Hydroxynonenal
LIP-1: Liproxstatin 1
MMF: Monomethyl fumarate
MS: Multiple sclerosis
Nrf2: Nuclear factor erythroid 2-related factor 2
OA: Osteoarthritis
OSC: Organotypic slice culture
PPMS: Primary progressive multiple sclerosis
RA: Rheumatoid arthritis
RRMS: Relapsing-remitting multiple sclerosis
SF: Synovial fibroblast
SIH: Salicylaldehyde isonicotinoyl hydrazone
SPMS: secondary progressive multiple sclerosis
