# Supplementary figures and images for: Fumarate-based drugs protect against neuroinflammation via upregulation of anti-ferroptotic pathways
Source: J Neuroinflammation. 2025 Oct 27;22:241. doi: 10.1186/s12974-025-03592-3 (PMC12560501; doi:10.1186/s12974-025-03592-3)

Figure 3c

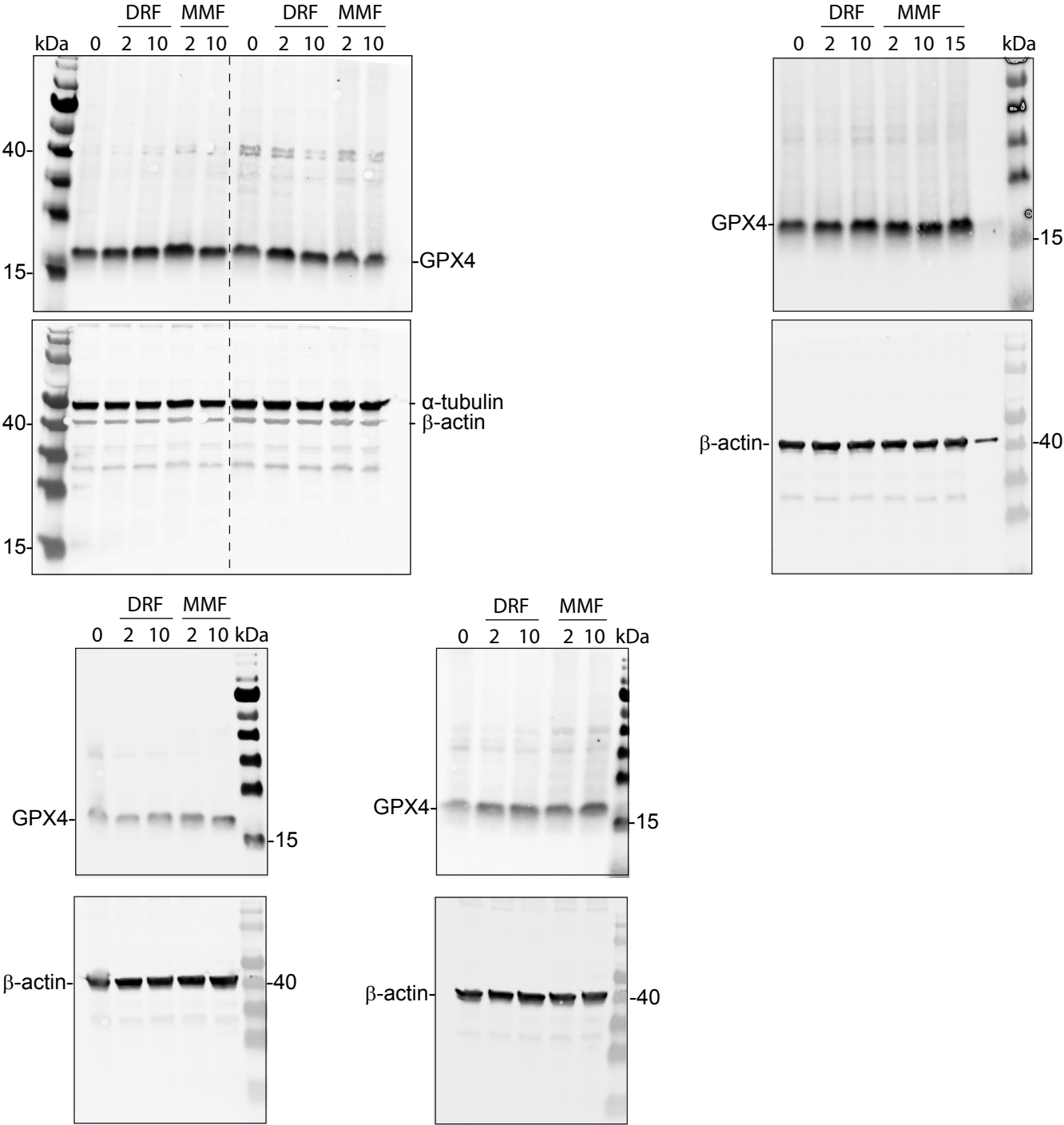

Figure 3d

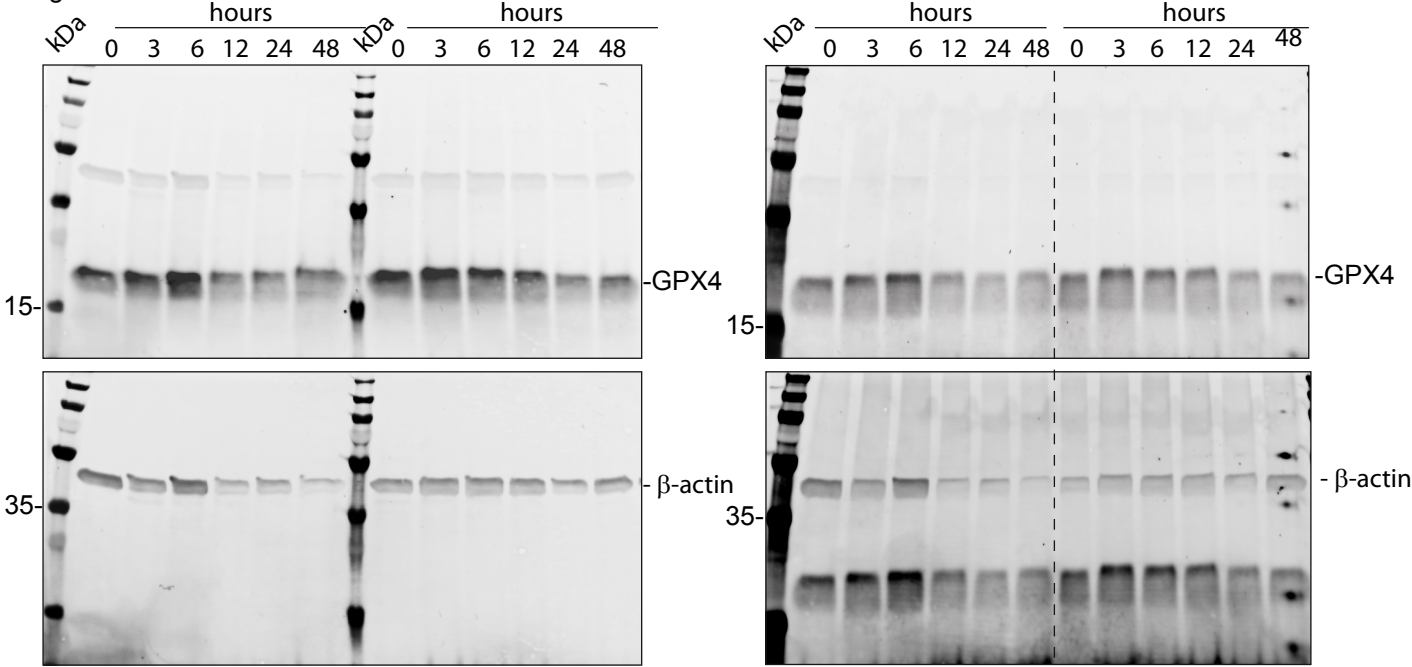

Supplement: Supplementary file 1 — Supplementary material 1. [file 12974_2025_3592_MOESM1_ESM.pdf]

Figure 5B

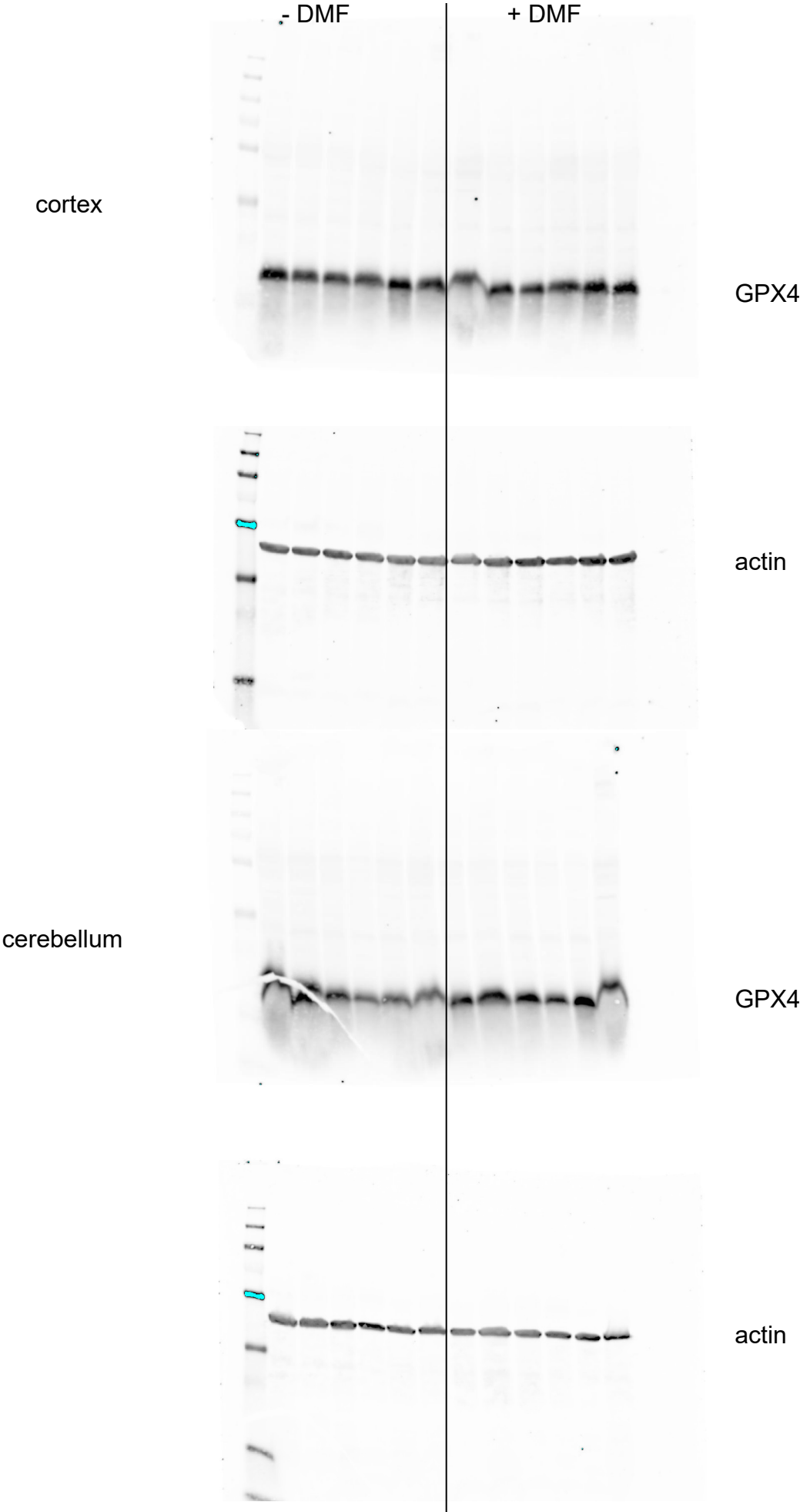

Supplement: Supplementary file 2 — Supplementary material 2. [file 12974_2025_3592_MOESM2_ESM.pdf]

Figure 6G, H

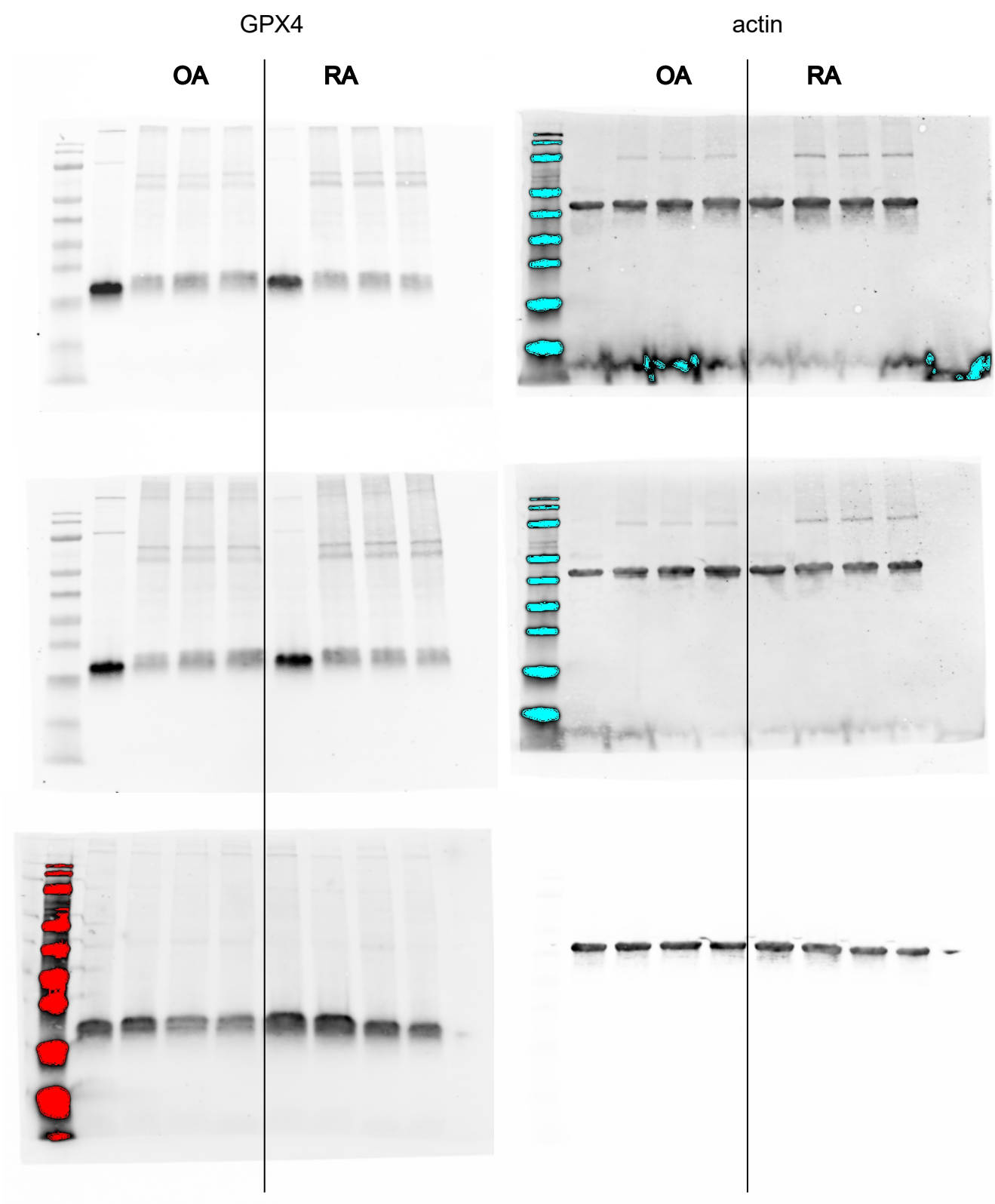

Supplement: Supplementary file 3 — Supplementary material 3. [file 12974_2025_3592_MOESM3_ESM.pdf]
